# Emergency department visits by nursing home residents. A retrospective Italian study of administrative databases from 2015 to 2019

**DOI:** 10.1186/s12877-024-04912-7

**Published:** 2024-03-28

**Authors:** Beatrice Albanesi, Alessio Conti, Gianfranco Politano, Valerio Dimonte, Maria Michela Gianino, Sara Campagna

**Affiliations:** 1 Department of Sciences of Public Health and Pediatrics, University of Turin, Via Santena 5 bis, Turin, 10126 Italy; 2 Department of Control and Computer Engineering, Politecnico di Torino, Turin, Italy

**Keywords:** Nursing home, Emergency Department, Emergency visits, Older adults, Retrospective study

## Abstract

**Background:**

Visits to Emergency Departments (ED) can be traumatic for Nursing Home (NH) residents. In Italy, the rate of ED visits by NH residents was recently calculated as 3.3%. The reduction of inappropriate ED visits represents a priority for National Healthcare Systems worldwide. Nevertheless, research on factors associated with ED visits is still under-studied in the Italian setting. This study has two main aims: (i) to describe the baseline characteristics of NH residents visiting ED at regional level; (ii) to assess the characteristics, trends, and factors associated with these visits.

**Methods:**

A retrospective study of administrative data for five years was performed in the Piedmont Region. Data from 24,208 NH residents were analysed. Data were obtained by merging two ministerial databases of residential care and ED use. Sociodemographic and clinical characteristics of the residents, trends, and rates of ED visits were collected. A Generalized Linear Model (GLM) regression was used to evaluate the factors associated with ED visits.

**Results:**

In 5 years, 12,672 residents made 24,609 ED visits. Aspecific symptoms (45%), dyspnea (17%) and trauma (16%) were the most frequent problems reported at ED. 51% of these visits were coded as non-critical, and 58% were discharged to the NH. The regression analysis showed an increased risk of ED visits for men (OR = 1.61, 95% CI 1.51–1.70) and for residents with a stay in NH longer than 400 days (OR = 2.19, 95% CI 2.08–2.31).

**Conclusions:**

Our study indicates that more than half of NH residents’ ED visits could potentially be prevented by treating residents in NH. Investments in the creation of a structured and effective network within primary care services, promoting the use of health technology and palliative care approaches, could reduce ED visits and help clinicians manage residents on-site and remotely.

## Background

Population aging is becoming a global economic public health problem [1–3]. In the European Union alone, the number of potentially care-dependent older adults is expected to rise, from about 30.8 million in 2019 to 38.1 million in 2050 [2]. Hence, an increase in the number of older adults who will need long-term residential care, such as Nursing Homes (NH), is foreseeable [3].

Emergency Departments (ED) play a pivotal role in the provision of care to NH residents by providing urgent care for an unexpected acute disease [4–6], or as an access point for hospitalization when there is a deterioration of a chronic disease [7]. Generally, NH residents represent the most fragile group of elderly who, when compared to their pair age-matched community cohorts, report poorer health status, higher rates of dementia, and more severe mental diseases [8–9]. Therefore, providing appropriate geriatric care is often a challenge within the ED environment [10]. NH residents are at increased risk of complications during transfer and attendance at ED [11]. ED visits can themselves lead to increased mortality, episodes of delirium, falls, ED-acquired infections, and more possibilities of aggressive treatment, which affect patient care and quality of life [10–12]. Furthermore, ED visits involve significant use of health resources: approximately 95% of transfers from NH are made by ambulance, causing long ED waits, which contributes to ED overcrowding and overwork of professionals [13–15]. Although several studies [10–19] have identified the inadequacy of ED visits and their possible reduction through better clinical management of residents within the actual NH, the number of ED visits from NH is still high [15–20]. Arendts and colleagues [21] in their systematic review, showed that the percentage of ED visits by NH residents represents 0.4–2.4% of all ED visits yearly. Furthermore, the number of visits varied between countries, showing different trends. Wang and colleagues [22] estimated that the proportion of ED visits by NH residents in the United States 2005–2008 was 1.9% of all ED visits. Comparable data were reported in a Swiss University Hospital, where ED visits by NH residents increased by about 50.1% in five years, from 1.5% of all ED visits in 2005 to 1.9% in 2010 [4]. More concerning data were found in northern Italy, where the percentage of calls from NH for emergency medical service for their residents represented 3.3% of all emergency calls [23].

Although reasons for ED visits are often underreported, the lack of consensus on the appropriateness of visits and specific care pathways for NH residents are among the leading causes of ED visits [5, 21–24]. In particular, the characteristics and factors related to ED visits remain unclear [5, 19, 24]. Hence, to enable NH residents to receive suitable care for their conditions and avoid inappropriate and potentially harmful hospitalizations, a better understanding is needed of the factors underlying transfer processes and ED visits [19, 25].

NH facilities in Italy include all people permanently in need of care due to a physical or mental disability [26]. NH facilities are part of the social insurance system, partially managed by the state National Healthcare Service and partially by the private sector [26, 27]. NH establishments are regulated at the regional level, and in the Piedmont region they are responsible for the clinical condition and care needs of non-self-sufficient elderly who cannot be cared for at home; they generally provide 24-hour long-term care (LCT), accommodation, and catering [28]. NH staff are nurses or healthcare assistants, and generally no physicians are employed, but the general practitioner (GP) is referred to for residents’ care management [28, 29]. If NH residents require care management, staff usually have two ways to manage the situation: the first is to contact the GP or the physician employed in the facility, if available. The second, if the resident shows acute symptoms and the GP is not promptly available, is to call an ambulance or to transfer the residents to ED [27–29]. Given the lack of studies evaluating ED visits by NH residents in the Italian context, it could be fundamental to evaluate the characteristics of ED visits by NH residents, their trends, and factors associated with these ED visits.

Therefore, this study set itself two main objectives: (i) to describe the baseline characteristics of NH residents who visit the ED in a large Italian region; (ii) to assess the characteristics, trends and factors associated with these visits.

## Methods

### Study design and setting

A retrospective study was conducted over five years from 2015 to 2019 among NH residents in the Piedmont region (Italy). Piedmont is the second largest region of Italy with a population of more than four million inhabitants over an area of 25,387 km [30]. In 2019, this region offered 41,360 residential and semi-residential care facilities (945 beds/100,000 inhabitants) [31]. All ED visits by NH residents during the study period were analysed.

### Study population

NH residents visiting ED were the study population. Specifically, the sample was represented by users of the state National Health Service. Residents in vegetative state, minimum consciousness, locked-in syndrome, chronic neurological conditions (e.g., amyotrophic lateral or multiple sclerosis), or end-of-life/terminal condition were excluded.

### Data sources and procedures

Data were directly requested by the research team to the Epidemiology Unit of the Regional Health Service of the Piedmont Local Health Unit. Subsequently, the Epidemiology Unit extracted anonymised data based on requests made by the research team. These were aggregated while maintaining the confidentiality of individual-level information. The aggregated data was obtained by two ministerial administrative sets of electronic health records in the official Italian Information System [32] a country-wide health database in the NH residents’ information system (named Flows of Residential Services – FAR); and a database on admissions and use of the hospital ED (named C2 registry). The FAR collects and monitors quarterly information on Italian public residential and out-services and includes data on dispensing facilities and residents (such as the number of beds, sociodemographic and clinical information, clinical procedures delivered, admissions, and discharge information). The C2 registry provides monthly information on ED services and use. Data from these databases were merged into one single database and analysed as aggregated by one of the researchers (GMP).

### Variables and data organization

The data were merged by the research team into one single database, containing the following: (i) baseline characteristics (sociodemographic and clinical) of residents at the admission to NH; (ii) type of care provided; (iii) characteristics of ED visits. The sociodemographic and clinical characteristics of the residents included: sex and age; prevalent diagnosis at admission to NH; level of assistance intensity; level of independence expressed as activities of daily living (ADL); mobility; level of cognitive impairment and presence or absence of behavioural disorders; and length of stay in NH. Data were organized as follows: age was stratified into four classes (< 65; 66–80; 81–90; >91 years) or calculated as median and interquartile range. Clinical characteristics such as the prevalent diagnosis at admission to NH were organised according to the 9th International Classification of Diseases (ICD-9) revision [33] and grouped according to the frequency with which residents were admitted to NH. We included only the primary diagnosis assigned to each resident at admission to the NH. Specifically, we organised the diagnoses into the following groups: mental disorders; cardiovascular diseases; neurological diseases; endocrine diseases; residuals of trauma; musculoskeletal diseases; respiratory diseases; neoplasms; aspecific diseases; urogenital diseases; digestive diseases; perinatal or congenital disorders; hematological diseases; infectious diseases. The type and intensity of care provided to NH residents are regulated at a regional level depending on their functional, cognitive, and behavioral status, as well as their social condition, and the provision of care is based on a multidimensional evaluation made by a multidisciplinary commission [34]. This commission certifies three classes of intensity of care (low, medium, and high), depending on the complexity of care to be delivered to NH residents. In this study, the intensity of care was aggregated in two classes: ‘medium-low’ (comprising low and medium intensity of care) and ‘high’ (comprising high and very high intensity of care). The ‘medium-low’ class comprised care interventions with a moderate level of intensity and that require a lower complexity care intervention. ‘High’ class included care interventions with a higher intensity of care of residents with greater complexity of care. The ADLs were coded in the database as three levels of autonomy (totally dependent, partially dependent, and independent), as was the degree of mobility of residents (bedridden, dependent, and independent). Cognitive impairments were classified into three classes: severe, moderate, and mild; behavioural disorders were only classified as present or absent. All variables considered were classified according to the evaluation proposed in the FAR technical report [35].

The length of stay in NH represents the number of days that residents spent from admission to NH to access the ED, and was classified into two levels (< 400 and ≥ 400 days) based on the median value.

Data on ED visit characteristics included: applicant (emergency medical service intervention, family decision, transfer from a private or public institution, and physician’s decision); mode of arrival (public or private/own services, or unreported/unknown); time of arrival (in 2 time slots: 7am–8pm, and 9pm–6am); triage emergency code assigned at arrival (very critical, critical, not very critical and non-critical); the main symptom reported by NH residents at the ED triage, classified into the following: dyspnea, trauma, neurological symptoms, abdominal pain, fever, genitourinary symptoms, chest or thoracic pain, not traumatic bleeding, cardiac rhythm alteration, ocular disorders, shock or allergic reaction, dermatological symptoms and dentistry disorders; diagnosis at discharge from ED (coded by ICD-9); and lastly, destination after discharge from ED (returned to NH, admitted to hospital, dead in ED, and refused hospitalization).

### Data analysis

In line with our study aims, we performed a primary descriptive analysis on (i) baseline characteristics (sociodemographic and clinical) of residents at the admission to NH; (ii) characteristics of the total ED visits made by residents who visited the ED during the study period; (iii) symptoms reported at ED visits; and (iv) destination after visiting the ED. Categorical variables were shown as absolute frequencies and percentages, continuous variables as means, and standard deviation (SD). Median and interquartile range (IQR) were used when appropriate. Each investigated variable was summarised using a multiclass contingency table based on its frequency. A heat map [36] was used to visualize the associations between destination frequency after visiting the ED with the triage code received by the resident at the ED visit.

To assess the trends of ED visits, a fixed-effects regression analysis with a likelihood ratio test on panel data was used by the prevalent diagnosis of admission of residents to NH. This approach facilitated a detailed examination of temporal trends in ED visits over time. For the assessment of factors associated with ED visits, we used a Generalized Linear Model (GLM) with a Poisson bias function. As per the GLM principles [37], the probability was considered as the likelihood of residents visiting the ED at least once during the study period, with a dependent variable represented as a dummy variable (0 for no visits, 1 for visits). The resulting number represented the cumulative likelihood of access to ED by residents. To explore possible associations, the probability of accessing the ED served as the dependent variable, coupled with independent variables as the baseline characteristics of residents at NH admission, including age, sex, prevalent diagnosis in NH, the intensity of care, ADL, mobility, cognitive impairment, behavioural disorders, and length of stay in NH in days. The GLM was mutually adjusted for each independent variable to understand the factors influencing the probability of ED admission during the observation period and to assess the presence of confounders. In essence, the dependent variable represented the probability of making an ED visit by NH residents, and the analysis explored how the various independent variables influenced this probability within the observation period. The probability of access was expressed as an odds ratio (OR) with a 95% confidence interval (CI). Hence, the probability of accessing the ED was used as an indicator to explore and describe the use of ED among this population. For the fixed-effects and GLM analyses, missing data were excluded to ensure the integrity and coherence of the data. All analyses were performed with the software statistical package R [38]. Findings are reported as per Strengthening the Reporting of Observational Studies in Epidemiology (STROBE) guidelines [39] for observational studies of routinely collected data.

### Ethics

The Italian National Information System databases include the Flows of Residential Services and the C2 registry, which are official anonymised Ministerial Health information systems. All resident information is centrally anonymised and available to authorised institutions to be used for epidemiological and/or health organisation studies without any further authorisations. Therefore, approval from the ethics committee was not required and unnecessaty according to national regulations. Personal data treatments are carried out in compliance with the current rules set out in EU Regulation 2016/679 [40] and the current legislation on the Protection of Personal Data set out in Legislative Decree 101/2018 [41], as well as Legislative Decree 196/2003 [42] and subsequent amendments and additions. In particular, our study used statistical and aggregate data that were shared according to the current deontological rules for the processing of data from the National Statistical System for scientific research purposes, by the provisions of art. 5 ter of Legislative Decree 33 /2013 [43] as amended by Legislative Decree 97/2016 [44] and Legislative Decree 101/2018 [45]. For these reasons, a direct informed consensus could not apply to this study. However, adherence to Italian and European regulations was consistently maintained at all stages of the investigation.

## Results

A total of 24,208 residents were admitted to NH in the Piedmont Region from 2015 to 2019. During the study period, 12,672 residents of all 24,208 residents admitted to NH in the Piedmont Region, made 24,609 ED visits. These represented 1.02 ED visits per NH resident overall, and an average of 1.94 ED visits per resident per NH who was sent to ED during the study period. Among NH residents (Table 1), 72.1% were female and 27.9% were male. Most of these (48.6%) were aged 81 to 90 years, with a median age of 86 years (IRQ 80–90). The leading diagnosis at the time of admission to the NH were mental disorders (26.0%), cardiovascular disorders (23.1%), and neurological disorders (14.9%). Most of the residents received a high-intensity level of care (67.4%), were totally dependent for ADL (50.3%), and were bedridden (58.7%). About 42% had severe cognitive impairment and behavioural disorders (47.8%) and more than half (54.5%) had a length of stay of over 400 days, with a median of 465 (IRQ 194–904).

**Table 1:** Baseline characteristics of all 24,208 residents admitted to NH in the Piedmont Region, Italy, 2015–2019

Baseline Characteristics (*N* = 24,208)	*n*	%
**Sex**		
Female	17,462	72.1
Male	6,746	27.9
**Age**		
< 65	728	3.0
66–80	5,692	23.5
81–90	11,767	48.6
> 91	6,021	24.9
**Diagnosis at the admission in NH**		
Mental disorders	6,301	26.0
Cardiovascular diseases	5,586	23.1
Neurological diseases	3,601	14.9
Endocrine diseases	1,133	4.7
Residuals of trauma	607	2.5
Musculoskeletal diseases	586	2.4
Respiratory diseases	538	2.2
Neoplasms	467	1.9
Aspecific diseases	232	1.0
Urogenital diseases	215	0.9
Digestive diseases	193	0.8
Perinatal or congenital disorders	95	0.4
Hematological diseases	76	0.3
Infectious diseases	75	0.3
Missing	4,503	18.6
**Intensity of care**		
High–intensity	16,306	67.4
Medium–low intensity	7,902	32.6
**Activities of daily living**		
Totally dependent	12,172	50.3
Partially dependent	7,038	29.1
Independent	941	3.9
Missing	4,057	16.7
**Mobility**		
Bedridden	14,220	58.7
Assisted	3,381	14.0
Independent	2,550	10.5
Missing	4,057	16.8
**Cognitive impairment**		
Severe	10,152	41.9
Moderate	5,986	24.7
Mild	4,013	16.5
Missing	4,057	16.7
**Behavioural disorders**		
Present	11,574	47.8
Absent	8,577	35.4
Missing	4,057	16.8
**Length of stay in NH in days**		
≥ 400	13,203	54.5
< 400	11,005	45.5

NH: Nursing Homes; ED: Emergency Department;

Approximately 80% of ED visits were made through the intervention of emergency medical services, while 10.9% were by family decision (Table 2). Almost all ED visits (81.1%) occurred between 7am and 8pm. Most ED visits received a ‘not very critical’ emergency code (50.1%), for aspecific symptoms (45.3%), dyspnea (16.9%), trauma (16.3%) and neurological symptoms (5.7%). After ED visits, the majority of residents returned to NH (55.7%), 37.2% were admitted to the hospital, and 5.0% died in ED.

**Table 2:** Characteristics of 24,609 total ED visits made by 12,672 residents in the Piedmont region, Italy, 2015–2019

Characteristics of ED visits (*N* = 24,609)	*n*		%
**ED applicant**			
Emergency medical service	19,641		79.8
Family decision	2,679		10.9
Transfer from private or public institution	2,047		8.3
Physician’s decision	242		1.0
**Mode of arrival at ED**			
Public ambulance	19,847		80.6
Private/own service	4,018		16.3
Unreported/unknown	744		3.0
**Time of arrival in ED**			
7am to 8pm	19,966		81.1
9pm to 6am	4,643		18.9
**Triage emergency codes**			
Very critical	2,235		9.1
Critical	9,778		39.7
Not very critical	12,329		50.1
Non-critical	267		1.1
**Symptoms reported at triage**			
Aspecific symptoms	11,146		45.3
Dyspnea	4,162		16.9
Trauma	4,010		16.3
Neurological symptoms	1,421		5.8
Abdominal pain	1,120		4.6
Fever	896		3.6
Genitourinary symptoms	702		2.9
Chest or thoracic pain	353		1.4
Bleeding (not traumatic)	321		1.3
Cardiac rhythm alteration	236		1.0
Ocular disorders	145		0.6
Shock or allergic reaction	40		0.2
Dermatological symptoms	40		0.2
Dentistry disorders	17		0.1
**Diagnosis at discharge from the ED**			
Trauma	4,913		20.0
Respiratory diseases	4,807		19.5
Aspecific diseases	4,037		16.4
Cardiovascular diseases	2,373		9.6
Digestive diseases	1,429		5.8
Urogenital diseases	1,312		5.3
Endocrine diseases	742		3.0
Hematological diseases	718		2.9
Neurological diseases	711		2.9
Perinatal or congenital disorders	667		2.7
Mental disorders	605		2.5
Musculoskeletal diseases	547		2.2
Infectious diseases	521		2.1
Neoplasms	93		0.4
Missing	1,134		4.6
**Destination after discharge from the ED**			
Returned to NH	13,703		55.7
Admitted to the hospital	9,172		37.3
Dead in the ED	1,204		4.9
Refused hospitalization	530		2.2

NH: Nursing Homes; ED: Emergency Department

At the ED visit (Table 3), NH residents most frequently reported aspecific symptoms, which got the majority of non-critical (25.4%) and critical (19.9%) triage emergency codes. Trauma was higher among the non-critical ED codes (11.5 VS 4.8%), while dyspnea was most frequently reported among those who received a critical ED code (13.0% VS 4.0%).

**Table 3:** Reported symptoms, stratified by emergency codes, of the 24,609 total ED visits made by 12,672 NH residents in the Piedmont region, Italy, 2015–2019

Reported symptoms at the ED visits (*N* = 24,609)	Emergency code					Total reported symptoms in the ED	
	Critical			Not critical			
	*n*	(%)		*n*	(%)	*N*	(%)
Aspecific symptoms	4890	(19.9)		6256	(25.4)	11,146	(45.3)
Dyspnea	3189	(13.0)		973	(4.0)	4162	(16.9)
Trauma	1173	(4.8)		2837	(11.5)	4010	(16.3)
Neurological symptoms	1073	(4.4)		348	(1.4)	1421	(5.8)
Abdominal pain	458	(1.9)		662	(2.7)	1120	(4.6)
Fever	437	(1.8)		459	(1.9)	896	(3.6)
Chest or thoracic pain	244	(1.0)		109	(0.4)	353	(1.4)
Cardiac rhythm alteration	175	(0.7)		61	(0.2)	236	(1.0)
Bleeding (not traumatic)	155	(0.6)		166	(0.7)	321	(1.3)
Genitourinary symptoms	143	(0.6)		559	(2.3)	702	(2.9)
Shock or allergic reaction	30	(0.1)		10	(0.01)	40	(0.2)
Ocular disorders	27	(0.1)		118	(0.5)	145	(0.6)
Dermatological symptoms	13	(0.05)		27	(0.1)	40	(0.2)
Dentistry disorders	6	(0.02)		11	(0.04)	17	(0.1)
	12,013	(48.9)		12,596	(51.1)	24,609	(100)

NH: Nursing Homes; ED: Emergency Department

**Fig. 1:**
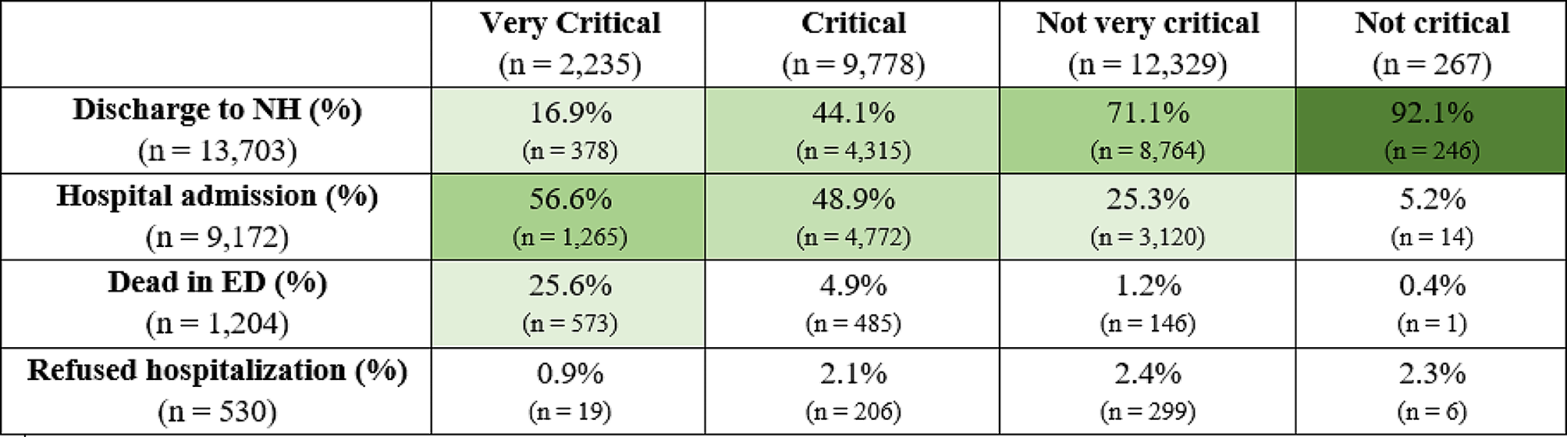
Destination frequency after visiting ED, stratified by emergency codes, of the 24,609 total ED visits made by 12,672 NH residents in the Piedmont region, Italy, 2015–2019

More than half of NH residents who received a very critical code were admitted to hospital (56.6%), while one in four (25.6%) died. Among NH residents who received a critical code, 48.9% were admitted to the hospital and 44.1% returned to NH. The majority (71.1%) of NH residents who received a not very critical code at the ED visit returned to NH, and one in four (25.3%) were admitted to the hospital. Almost all (92.1%) of residents who received a non-critical code returned to NH (Fig. 1).

### Trends in ED visits sorted by residents’ diagnoses on admission to NH

**Fig. 2:**
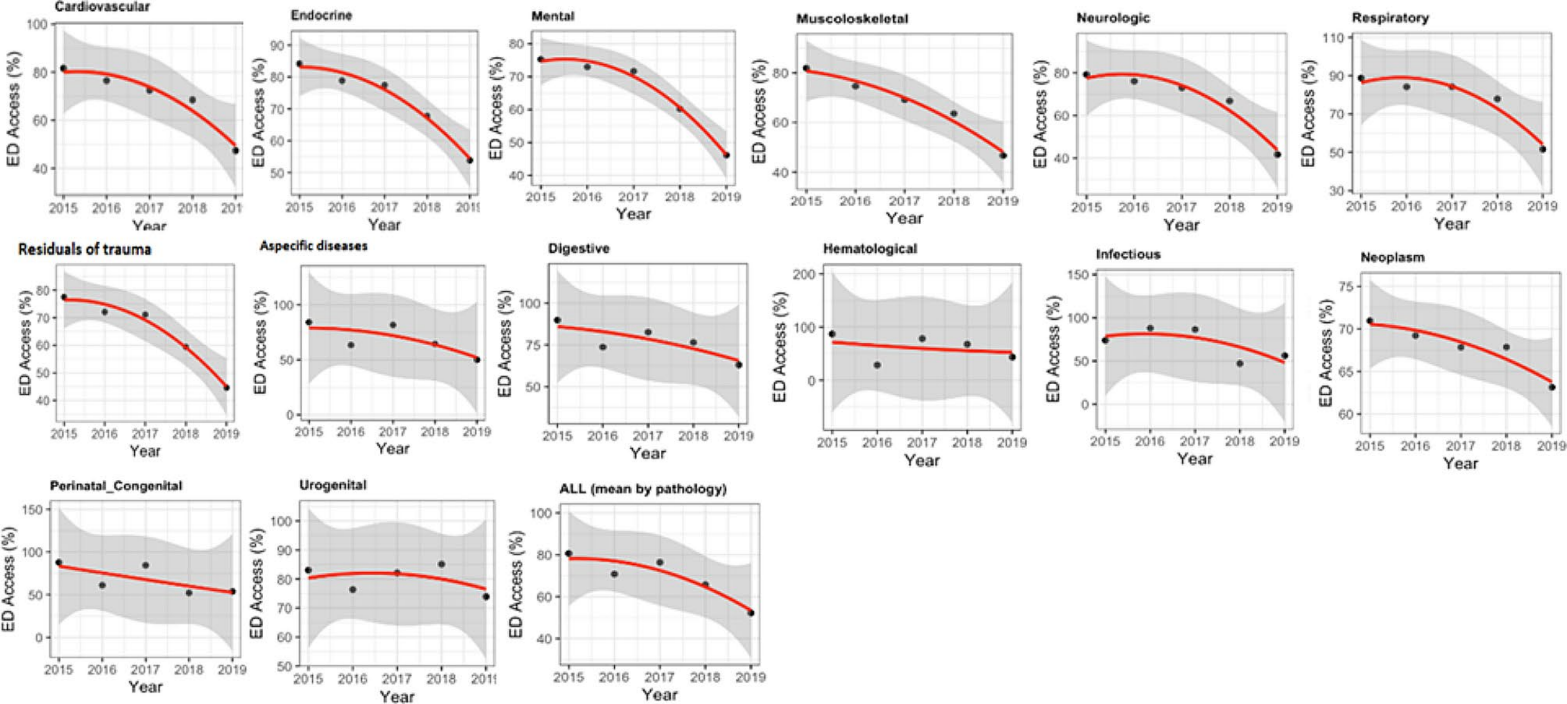
Trends of the total 24,609 ED visits made by 12,672 NH residents, classified according to the prevalent diagnosis at the admission to NH in the Piedmont region, Italy, 2015–2019

All trends in ED visits, classified by residents’ diagnoses on admission to NH, showed a decrease during the study period (Fig. 2). Among these, cardiovascular, endocrine, mental, musculoskeletal, neurologic, respiratory, and residuals of trauma had a marked reduction. By contrast, aspecific diseases, digestive, hematologic, infectious, neoplasms, perinatal or congenital disorders, and urogenital diseases showed only a slight reduction. These findings were confirmed by the general regression model, showing a significant linear trend (b = -6.1956, *p* = 0.0439, 95% CI = [-12.0707, -0.3204]), combined with a non-significant coefficient for quadratic terms (b = − 1.6802, *p* = 0.3783, 95% CI = [-8.1199, 4.7595]).

### Factors associated with ED visits

The regression analyses (Table 4) showed a significantly increased probability of ED visits for men (OR = 1.61, 95% CI 1.51–1.70) and for residents whose stay in NH was longer than 400 days (OR = 2.19, 95% CI 2.08–2.31). Residents admitted to NH for mental disorders (OR = 0.67, 95% CI 0.52–0.85), residuals of trauma (OR = 0.68, 95% CI 0.51–0.89) and neoplasms (OR = 0.73, 95% CI 0.55–0.98) had a significantly reduced probability of ED visits. Similarly, bedridden residents (OR = 0.77, 95% CI 0.69–0.85), those with severe cognitive impairment (OR = 0.77, 95% CI 0.71–0.83) and no behavioural disorders (OR = 0.87, 95% CI 0.83–0.92) had a significantly lower probability of ED visits. Residents aged between 81 and 90 years (OR = 0.80, 95% CI 0.68–0.93) or > 91 (OR = 0.59, 95% CI 0.50–0.69) had a reduced probability of ED visits.

**Table 4:** Factors associated with the total 24,609 ED visits made by 12,672 NH residents in the Piedmont region, Italy, 2015–2019

Factors associated with ED visits (*N* = 24,609)	OR	[95% CI]
**Sex**		
Female	1	
Male	1.61*	[1.51–1.70]
**Age**		
< 65	1	
66–80	0.99	[0.84–1.15]
81–90	0.80*	[0.68–0.93]
> 91	0.59*	[0.50–0.69]
**Diagnosis at admission to NH**		
Aspecific diseases	1	
Mental disorders	0.67*	[0.52–0.85]
Residuals of trauma	0.68*	[0.51–0.89]
Neoplasm	0.73*	[0.55–0.98]
Musculoskeletal diseases	0.66	[0.57–1.00]
Perinatal or congenital disorders	0.71	[0.46–1.10]
Hematological diseases	0.72	[0.45–1.13]
Neurological diseases	0.72	[0.46–1.10]
Cardiovascular diseases	0.80	[0.63–1.02]
Endocrine diseases	0.84	[0.65–1.09]
Infectious diseases	0.90	[0.56–1.45]
Digestive system diseases	1.09	[0.76–1.56]
Respiratory diseases	1.24	[0.93–1.65]
Urogenital disease	1.28	[0.90–1.81]
**Level of assistance**		
Medium–low intensity	1	
High intensity	1.07	[1.00–1.13]
**Activities of daily living**		
Independent	1	
Totally dependent	0.87	[0.74–1.02]
Partially dependent	1.05	[0.91–1.22]
**Mobility**		
Independent	1	
Bedridden	0.77*	[0.69–0.85]
Assisted	1.05	[0.94–1.16]
**Cognitive impairment**		
Mild	1	
Severe	0.77*	[0.71–0.83]
Moderate	0.94	[0.87–1.02]
**Behavioural disorders**		
Present	1	
Absent	0.87*	[0.83–0.92]
**Length of stay in NH in days**		
< 400	1	
≥ 400	2.19*	[2.08–2.31]

NH: Nursing Homes; ED: Emergency Department; * *p*<0.05; Missing data were excluded from the analysis;

## Discussion

This study aimed to assess the characteristics, trends, and factors associated with ED visits by NH residents over a five-year period. Our sample was characterized by the high frequency of female residents, aged over 70 years, with a high-dependency profile, frequently showing behavioural disorders, cognitive impairment, and reduced mobility. Furthermore, our residents also had an extended stay in NH, often exceeding one year. These characteristics make our population comparable to other internationally reported data [18, 46, 47], in which NH residents are generally represented by a high prevalence of frail or pre-frail elderly people [47].

Aspecific symptoms and trauma were the main reasons reported by ED triage nurses, who coded them most frequently as non-critical situations, and mostly were generally discharged to NH. The higher frequency of aspecific symptoms, reported as the main problem leading to ED visits, might be explained by the gradual onset of functional decline and fragile status (e.g., poor general health, poor physical functioning), which typically characterizes NH residents [20]. Indeed, the progressive loss of autonomy, increased risk of immobility, and reduced cognitive performance clearly influences the health status of NH residents. This could exacerbate symptoms of existing conditions that may be difficult to attribute to a specific problem during triage [19]. Additionally, many NH residents are unable to report any changes in their health status or provide indications of the onset of new symptoms [48] and it is not unusual that on transfer to ED, residents commonly present information gaps and lack detailed clinical documentation from the NH, which may affect the admission diagnosis and assignment of ED code [49]. Therefore, it would be interesting to understand whether communication between the NH and the ED is lacking, or whether patients’ situations are not known, as a diagnostic hypothesis is difficult to formulate without a clinical diagnostic evaluation [50, 51].

Trauma was also highly frequent among non-critical emergency codes. Traumatic events have already been reported as one of the leading causes of ED visits among NH residents [52], mainly due to neurological or musculoskeletal conditions [52, 53]. Trauma could be associated with the routine moving of NH residents by nursing staff or healthcare assistants, which could lead to falls or injuries that require in-depth evaluation through ED visits [51–54]. Falls represented one of the main reasons for NH residents’ ED visits, responsible for 25–87% of ED visits or hospitalizations with at least one night’s stay [51, 54, 55]. Recently, one in four fall-related transfers from NH was rated potentially avoidable, by developing partnerships with outpatient clinics for imaging services, and strengthening geriatric expertise in NH through clinical training and advanced nurse practitioners [54].

Reasons contributing to inappropriate ED visits by NH residents may include health system factors and nurse/physician factors [48]. Concerning health system factors, the provision of ambulance transports to ED as part of the Italian emergency medical service [22] could have contributed to the high prevalence of this mode of arrival in our sample. Almost all ED visits during the day could be linked to a higher number of NH staff during day shifts [36], who may be more concerned about assessing residents with acute health conditions than night shifts, when staffing ratios are reduced [56, 57]. Regarding nurse/physician factors, staffing levels in NH have often been associated with inappropriate ED visits. Nursing shortages, combined with an increased workload in NH, lead to missed care for residents [56–58]. Delays in monitoring residents’ condition and rapid assessment by physicians can exacerbate situations that will be solved in NH by sending residents to ED [19, 48]. Providing NH with diagnostic and therapeutic services, such as radiology or consultation with a specialized physician, could decrease residents’ ED visits and reduce healthcare costs [59, 60]. Furthermore, increasing healthcare specialist consultations would facilitate timely treatment of residents before their clinical condition deteriorates to the point where ED visits are needed [57]. Finally, investments in new infrastructures (e.g., targeted telephone triage, apps, or tele-consultation), and promoting greater use of technology and telemedicine among NH staff, would limit the risks involved in referral to ED for frail residents [19, 61, 62]. Also, experiences of mobile ED to NH residents as an alternative to transferring residents, obtained positive results in the reduction of ED visits [63, 64]. On this same topic, a recent experience of a NH telehealth program on NH and LTC [62] obtained significant reductions in residents’ ED visits, hospitalizations, and spending.

ED ‘critical’ codes related to trauma are consistent with major injuries in residents returning to NH, such as hip fractures or post-fall intracranial injuries, deaths or disability [54]. Fear of falling can result in further loss of function, depression, feelings of helplessness, and social isolation [65]. Other critical emergency codes were assigned for dyspnea, and frequently resulted in hospitalization or death of NH residents [66]. Diagnosis of respiratory diseases such as asthma, pneumonia, or chronic obstructive pulmonary disease increases the risk of visiting ED for NH residents [19], since they necessitate treatments such as oxygen therapy or invasive mechanical ventilation. NH-acquired pneumonia (NHAP) is among the leading causes of hospitalization and mortality in NH residents, generally representing treatment challenges for ED physicians [67].

Deaths in ED could be avoided through interventions primarily directed toward those who would not benefit from any aggressive treatment [68]. Specifically, enhanced palliative care approaches promoted by end-of-life (EOL) communication [69] may reduce ED visits. Addressing advanced care planning discussions based on NH residents’ preferences or opinions could provide a solution to avoid ED visits by focusing on their quality of life rather than active/aggressive treatments [69, 70]. Early involvement of NH residents in decisions about their care and consideration of where this could be delivered would allow them to direct their own EOL care [68–72].

Overall, our results show that number of ED visits are progressively decreasing but considering that the prevalence of ED calls in the Piedmont region is at 3.3% [23], more efforts should be made to manage inappropriate situations. Unlike other studies [4–6, 11, 13, 17] showing increased ED visits among older residents of NH, our findings suggest that older age, as well as the presence of cognitive impairment, can act as protective factors for ED referral. Healthcare professionals may strive to preserve the residual quality of life of older residents, preferring to send those who are younger or less cognitively compromised to the ED [46].

A higher occurrence of ED visits was observed in men. Sex differences are discussed in literature without definitive practical guidance. Males seems to be at risk of developing severe pathological conditions and aging less actively, while women are more frequently alone and older, resulting in a decrease of concern among their caregivers and less pressure on healthcare professionals to send them to ED [73].

Unexpectedly, residents having stayed over 400 days in NH double their chance of visiting the ED. Previous studies examining the length of stay in NH showed that much depends on facilities, context, and directives [9, 72, 74]. In this case, improving palliative care and assuring early EOL conversations could help determine who will benefit from an ED visit [69].

Our results showed missing data on baseline characteristics at the time of admission of residents to NH, calling for careful consideration of the potential effect on the safety of care provided above any possible change in obtained findings. The lack of accuracy in clinical data collection is a well-known phenomenon in the international literature, especially in NH or LTC settings, that could be a cause of missing data [75]. It is plausible that there are underlying reasons for not documenting specific clinical variables, such as a shortage of administrative personnel, resource limitations, or improper documentation [75, 76]. Without complete and accurate data documentation, healthcare professionals may struggle to provide tailored care to NH residents, affecting their decisions to send them to the ED. To solve this problem, improving documentation practices, staff training, and technological solutions were possible interventions that showed positive results and should be implemented consistently in NH [75, 76].

### Limitations and strengths

The main limitation of this study is related to the use of administrative databases. Our study showed the presence of missing data. This limitation could preclude certain meaningful comparisons and may have influenced our results, compromising the accuracy of our conclusions. The lack of detail on the reason for referral to the ED, as well as the tests and interventions performed during and after the ED visit, limited our ability to assess the appropriateness of ED visits. Data on patients’ functional and cognitive status on ED arrival and discharge, presence of delirium or falls during the ED visit, or pharmacological treatment performed, were not available; neither were data on frequent ED users. An additional limitation of our study was the aggregated nature of the data received, which precluded our possibility of performing an analysis of person-time at risk. Furthermore, our study did not directly compare the visits of the ED with the overall trend of the ED visits. This could have limited the generalisation of our results. Finally, data are reported from a single institution in a specific geographical context of one state National Health system, limiting the generalizability of our findings to other healthcare systems. However, the inclusion of a large sample of NH residents over a five-year study period allowed the investigation of trends in ED visits, and this, combined with the systematic identification of each ED visit with its assigned emergency code, provided detailed information on the delivery of emergency services to this vulnerable population.

## Conclusion

Our study shows that some situations are potentially preventable by direct action in NH, and points to future fields for further research. Established community patterns of NH-to-ED referral could also help enhance care coordination for NH residents. Improved accessibility and continuity of community care is necessary to reduce ED visits by NH residents, as well as the utilization of available healthcare resources by shifting from hospital to long-term and community-based care. In Italy, the recent DM 77 2022 [77] offers the opportunity to make important changes to the current strategic coordination plan, with the institution of regional operative centres, the 116,117-call number [78] for the management of non-critical situations, and the provision of a continuity-of-care unit – a mobile district team for the management of people in particularly complex clinical and care conditions. Moreover, the planned future implementation of community hospital and telemedicine services could provide a further contribution to create a comprehensive network between ED and primary healthcare services. Furthermore, EOL and palliative care services are needed to improve residents’ remaining quality of life. Future research is needed to identify potentially multifactorial ED visits, by merging periodical administrative database flows and real-time clinical data from NH. Lastly, the conduction of longitudinal studies to deepen the understanding of the factors influencing ED visits among NH residents is warranted to enhance the comprehension of this phenomenon.

## Data Availability

The datasets used and/or analysed during the current study are available from the corresponding author on reasonable request.

## Abbreviations

NH: Nursing Homes
ED: Emergency Department
LCT: Long Term Care
GP: General Practitioner
FAR: Flows of Residential Services
ICD9: International Classification of Diseases 9 edition
ADL: Activities of Daily Living
SD: Standard Deviation
GLM: Generalized Liner Model
OR: Odds Ratio
CI: Confidence Interval
STROBE: Strengthening the Reporting of Observational Studies in Epidemiology
ID: Identify Number
IRQ: Interquartile Range
NHAP: Nursing Home Acquired Pneumonia
EOL: End of Life
